# Patient experience with subcutaneous immunoglobulin 20%, Ig20Gly, for primary immunodeficiency diseases: a prespecified post hoc analysis of combined data from 2 pivotal trials

**DOI:** 10.1186/s12865-020-00346-z

**Published:** 2020-05-04

**Authors:** Lisa M. Meckley, Yanyu Wu, Diane Ito, Todd Berner, Barbara McCoy, Leman Yel

**Affiliations:** 1 Shire US Inc., a Takeda company, Takeda Pharmaceutical Company Limited, 650 E Kendall St, Cambridge, MA 02142 USA; 2 Baxalta US Inc., a Takeda company, Cambridge, MA USA; 3 Baxalta US Inc., a Takeda company, Chicago, IL USA; 4 Baxalta Innovations GmbH, a Takeda company, Vienna, Austria

**Keywords:** Ig20Gly, Immunoglobulin, Subcutaneous, Satisfaction, Preference, Patient experience

## Abstract

**Background:**

Often, patients with primary immunodeficiency diseases (PID), which are marked by the absence or loss of functional antibodies, require lifelong treatment with immunoglobulin (IG) replacement therapy administered either intravenously (intravenous immunoglobulin [IVIG]) or subcutaneously (subcutaneous immunoglobulin [SCIG]). In patients with PID, the 20% SCIG product, Ig20Gly, was shown to be efficacious and well tolerated in 2 phase 2/3 trials conducted in North America and Europe. This analysis evaluated patient satisfaction with Ig20Gly therapy and treatment preferences.

**Methods:**

This prespecified post hoc analysis showed combined data from 2 Ig20Gly pivotal trials. Treatment satisfaction was assessed in the pre-Ig20Gly period and after ≥11 months of Ig20Gly treatment using the Life Quality Index (LQI; both studies) and the Treatment Satisfaction Questionnaire for Medication-9 (TSQM-9; North American study only). Treatment preference was assessed using a survey at the end of the European study. Median within-patient differences in LQI and TSQM-9 scores between the pre-Ig20Gly period and the end of the Ig20Gly treatment period were assessed using the Wilcoxon signed-rank test.

**Results:**

A total of 113 patients (*n* = 68 [North American]; *n* = 45 [Europe]) with PID were included in the analysis. In the combined LQI analysis (*n* = 110), significant improvements were observed in the *treatment interference* (median ∆: + 2.8; *P* = 0.006) and *therapy setting* (median ∆: + 5.6; *P* < 0.0001) domains, and in the item-level scores for *convenience* (median ∆: + 1.0; *P* < 0.0001) and *interference with work/school* (median ∆: + 1.0; *P* = 0.0001) categories. In the subgroup analyses, significant improvements in the *treatment interference* and *therapy settin*g domains and the *convenience* and *interference with work/school* items were observed for those who had previously received treatment outside the home, those who had previously received IVIG, and those in the North American study. Significant improvements were observed in the TSQM-9 *treatment convenience* domain (median ∆: + 11.1; *P* < 0.0001) and selected item-level scores in the North American study. In the European study, most (88.9%) patients preferred to continue Ig20Gly versus other IG treatments.

**Conclusions:**

After ≥11 months of taking Ig20Gly, patients reported high levels of treatment satisfaction, convenience, and preference for Ig20Gly, with consistent results across studies and use of multiple patient-reported outcome measures.

## Background

Primary immunodeficiency diseases (PID) comprise a heterogeneous group of >300 congenital disorders characterized by a genetic defect in the adaptive or innate immune system [1]. Among the 6.2 million individuals affected worldwide [2], primary antibody deficiencies represent the most prevalent PID diagnosis, making up nearly 50% of cases [3]. Patients with PID are susceptible to recurrent, serious bacterial infections (SBIs) and, without appropriate management, can experience long-term sequelae, such as bronchiectasis, autoimmune and gastrointestinal disorders, and progressive lung disease. These complications, in turn, can adversely affect the patient experience, including social relationships and health-related quality of life (HRQoL), daily work, and school activities [4–9].

Immunoglobulin (IG) replacement therapy (IGRT) is the mainstay of treatment for PID marked by the absence or loss of functional antibodies, including agammaglobulinemia, common variable immune deficiency, specific antibody deficiency, and immunoglobulin G (IgG) subclass deficiencies [10]. Clinical evidence indicates that IG administered intravenously or subcutaneously in patients with PID is well tolerated, significantly reduces the frequency of infections, and may enhance HRQoL [11–19]. Effective treatment, however, typically requires intravenous IG (IVIG) or facilitated subcutaneous IG (SCIG) infusions given every 3 to 4 weeks [20], or conventional SCIG infusions administered more often (from daily to every 2 weeks) [11]. Because IGRT is lifelong and patient satisfaction is a key factor in adherence [21], researchers and healthcare providers have considerable interest in identifying methods for improving the treatment experience for patients.

CUVITRU (Ig20Gly; immune globulin subcutaneous [human] 20%, Baxalta US Inc., a Takeda company, Westlake Village, CA, USA) is a ready-for-use, sterile liquid preparation of highly purified, concentrated IgG antibodies that is approved in the United States and Europe for the treatment of PID in adults and children aged ≥2 years [22, 23]. Because higher IG concentration reduces the infusion volume required, Ig20Gly treatment may enable shorter infusion duration compared with less concentrated SCIG products [12]. Two phase 2/3 multicenter, open-label clinical trials demonstrated the efficacy, pharmacokinetics, tolerability, and safety of Ig20Gly treatment in patients with PID in North America and Europe [11, 12]. The objective of this analysis was to understand the patient experience through the patients’ treatment satisfaction and preferences with Ig20Gly therapy during the registration pivotal trials.

## Methods

### Study design

This prespecified post hoc analysis of data from the Ig20Gly phase 2/3 pivotal trials was conducted in North America and Europe (NCT01218438 and NCT01412385).

The methods for these studies have been published previously [11, 12]. Briefly, in period 1 (pre-Ig20Gly period) of the 4-period North American study, patients were treated with IVIG 10% administered at 3- or 4-week intervals for 13 weeks and then switched to weekly Ig20Gly infusions for periods 2 to 4. Ig20Gly doses adjusted to 145% of the IVIG dose were administered for 12 to 16 weeks in period 2 and for 12 weeks in period 3. Patients were treated with individually adapted Ig20Gly doses for 40 weeks in period 4 [12].

In period 1 (pre-Ig20Gly period) of the 2-period European study, patients were treated with weekly SCIG 16% for 12 weeks or IVIG 10% every 3 or 4 weeks for 13 weeks. Patients were assigned to the same route of administration that was used prior to entering the study. In period 2, patients were switched to weekly Ig20Gly infusions administered at a weekly equivalent dose for approximately 12 months [11]. Both studies complied with the Declaration of Helsinki and the international standards of Good Clinical Practice [11, 12].

### Study setting

The North American study included patients from 15 sites in the United States and Canada; 96% infused Ig20Gly at home [12]. The European study was conducted at 16 sites in 7 countries (Germany, Austria, Sweden, United Kingdom, Netherlands, Belgium, and Hungary), with 95.8% of patients receiving ≥1 Ig20Gly infusion at home and 74.1% (1740 of 2349) of all Ig20Gly infusions being administered at home [11].

### Patient inclusion/exclusion criteria

Inclusion criteria for the North American and European studies have been previously described [11, 12]. Briefly, both studies included patients aged ≥2 years at screening with a documented diagnosis of PID requiring IGRT, a serum IgG trough level >500 mg/dL at screening, and no SBIs ≤3 months before screening [11, 12]. Included patients received a stable monthly mean IgG dose equivalent to 300–1000 mg/kg for ≥12 weeks before the first study treatment with Ig20Gly.

In the patient-experience analyses, additional exclusionary criteria were applied. For these analyses, patients were required to have ≥11 months of data in the Ig20Gly treatment phase (periods 2–4 in the North American study or period 2 in the European study) and no missing data at the end of the respective Ig20Gly treatment periods.

### Outcomes

#### Overview

Outcomes included summary domain scores, scores for prespecified selected items of the Life Quality Index (LQI; North America and Europe) and the Treatment Satisfaction Questionnaire for Medication-9 (TSQM-9; North America only), and responses to a treatment preference questionnaire (Europe only) (Table 1). This analysis used responses after treatment with Ig20Gly for all instruments from the end-of-study visit (or early-termination visit). In addition, the responses to the LQI and TSQM-9 were analyzed in the pre-Ig20Gly period (IVIG only in the North American study and either IVIG or SCIG in the European study).

**Table 1:** Patient-reported outcomes collected in the Ig20Gly North American and European phase 2/3 trials

Instrument	Domain score assessed	Concept	Specific item-level score assessed	Study included	
				North American	European
LQI^a^	*Treatment interference* (scaled score range: 0–100)	• Interference with social/family life • Time waiting • Treatment is worthwhile • Dependency on others • Freedom to take trips or move • Scheduled according to patient’s convenience	*Scheduled according to patient’s convenience* (score range: 1–7)	x	x
	*Therapy-related problems* (scaled score range: 0–100)	• Convenience • Painfulness • Health improvement • Anxiety or nervousness	*Convenience* (score range: 1–7)	x	x
	*Therapy setting* (scaled score range: 0–100)	• Interference with work/school • Given in a comfortable place • Given in a pleasant atmosphere	*Interference with work/school* (score range: 1–7)	x	x
TSQM-9^b^	*Effectiveness* (score range: 0–100)	• Ability of the medication to prevent or treat conditions • The way the medication relieves symptoms • Time before the medication works	–	x	–
	*Convenience* (score range: 0–100)	• Easy/difficult to use the medication in its current form • Easy/difficult to plan when to use the medication each time • Convenient/inconvenient to take the medication as instructed	*Convenient/inconvenient to take medication as instructed* (score range: 1–7)	x	–
	*Global satisfaction* (score range: 0–100)	• Overall confidence that taking this medication is a good thing • Certainty that good things about medication outweigh bad things • Overall satisfaction/dissatisfaction with medication	*Overall satisfaction/dissatisfaction with medication* (score range: 1–7)	x	–
Treatment preference^c^	–	• Like/dislike aspects of administration • Preference to continue Ig20Gly	Multiple items	–	x

^a^In the European study, the LQI was self-administered by patients aged ≥14 years (observer: patient) and completed by parents/caregivers for patients aged 2–13 years (observer: parent/caregiver). In the North American study, the LQI was self-administered by patients aged ≥13 years (observer: patient) and completed by parents/caregivers for patients aged 2–12 years (observer: parent/caregiver)

^b^In the North American study, the TSQM-9 was self-administered by patients aged ≥13 years (observer: patient) and completed by parents/caregivers for patients aged 2–12 years (observer: parent/caregiver)

^c^In the European study, the treatment preference questionnaire was self-administered by patients aged ≥14 years (observer: patient) and completed by parents/caregivers for patients aged 2–13 years (observer: parent/caregiver).

Ig20Gly, immune globulin subcutaneous (human) 20%: LQI, Life Quality Index; TSQM-9, Treatment Satisfaction Questionnaire for Medication-9

In the North American study, the LQI and TSQM-9 were completed by an observer (parent/caregiver) on behalf of a patient aged 2–12 years or by the patient if 13 years or older [12]. In the European study, the LQI and treatment-preference questionnaire were completed by an observer (parent/caregiver) on behalf of a patient aged 2–13 years or by patients if 14 years or older [11].

### Treatment satisfaction

#### Life quality index

The LQI is a 15-item validated measure assessing IG treatment satisfaction in the following 4 domains: (1) *treatment interference*, (2) *therapy setting*, (3) *therapy-related problems*, and (4) *cost*. Item scores in each domain are summed to derive a summary score for the respective domain. The range of possible scores varies by domain; however, all domain scores were scaled from 0 to 100 in this analysis. Higher scores indicated higher satisfaction (Table 1) [11, 12].

The present study analyzed summary scores for 3 domains (*treatment interference*, *therapy-related problems*, and *therapy setting*) and the individual item scores as follows: (1) “*scheduled according to patient’s convenience*” (*treatment interference*); (2) “*convenience*” (*therapy-related problems*); and (3) “*interference with work/school*” (*therapy setting*). For these 3 LQI domains, both raw and summary scale scores were calculated. At the item level, higher scores indicated higher satisfaction.

The individual LQI items assessed in this study were selected a priori by the authors based on each item’s likelihood of being impacted by the route of IGRT administration. The *cost* domain was not analyzed because patients did not pay for treatments in these studies. Furthermore, the psychometric validation study for the LQI recommended that the questions related to costs should be treated with caution because the lack of cost transparency and the different health systems did not allow the patient to assess therapy costs [24].

#### Treatment satisfaction questionnaire for medication

The TSQM-9 is a self-administered, 9-item, validated measure that assesses treatment satisfaction in the following 3 domains: *effectiveness*, *convenience*, and *global satisfaction*. The present study analyzed the TSQM-9 summary domain scores and individual scores for 1 item each in the domains of *convenience* (“*convenient/inconvenient to take medication as instructed*”) and *global satisfaction* (“*overall satisfaction/dissatisfaction with medication*”). Domain scores ranged from 0 to 100; higher scores indicated higher patient satisfaction (Table 1). For the 2 selected items, patients rated their satisfaction using a 7-point Likert scale, with higher scores indicating higher patient satisfaction.

#### Treatment preference

At the end-of-study visit, patients in the European study completed a nonvalidated survey, developed as a part of the clinical trial protocol. The survey assessed patient preference to continue Ig20Gly treatment versus previous treatment. Additional preferences evaluated were: site of treatment (home, hospital, or other), frequency of administration, number of needle sticks per month, total time spent on treatment per month, ease of administration, potential to self-administer, ability to fit the treatment into personal schedule, overall convenience, time required, complexity of administration, and the ability to administer with no supervision.

### Statistical methods

Continuous variables were summarized descriptively, using nonparametric methods (median, interquartile range). Categorical variables were expressed as a frequency (N) and percentage of total (%). The median within-patient differences (median ∆) in LQI and TSQM-9 scores between the end of the pre-Ig20Gly period and the end of the Ig20Gly treatment period were calculated. The differences in distributions of LQI and TSQM-9 scores between the pre-Ig20Gly period and the end of the study were assessed using the Wilcoxon signed-rank test. Therefore, the distribution of scores can have a statistically significant difference even if the median ∆ is zero. Subgroup analyses were conducted to test for differences in the LQI and TSQM-9 scores between patients categorized by age group (2–17 years, ≥18 years), prior route of IG administration (IV, SC), and site of prior treatment (home, other setting [doctor’s office, hospital, or infusion center]). The LQI was analyzed separately for the North American and European studies, with subgroups by prior route of administration also analyzed. In the European study, treatment preference at the end-of-study visit was analyzed overall and by age group, previous route of administration, and treatment setting. Statistical analyses were performed using SAS 9.3 (SAS Institute, Cary, NC, USA). A 2-sided *P* value < 0.05 was statistically significant.

## Results

To briefly summarize the original trials, 49 subjects included in the European study [11] and 77 subjects included in the North American study [12] received Ig20Gly. The European study population was 61.2% male, with a median age of 17.0 years (range: 2–67 years) and a median weight of 63.0 kg (range: 12.9–140.0 kg) [11]. The North American study population was 51.9% male, with a higher median age compared with that of the European study (36 years, range: 3–83 years), and median weight of 68.2 kg (range: 13.2–161.8 kg) [12]. In both trials, the median for completion of an infusion was 0.95 h and a median of 2 infusion sites was used [11, 12]. In the European study, the median maximum infusion rate was 20 mL/hour/site (range: 2.5–60.0 mL/hour/site) and the median infusion volume was 16.6 mL/site (range: 6.5–48.0 mL/site) [11]. In the North American study, the maximum infusion rate per site (60 mL/h/site [range: 4.4–180.0 mL/hour/site]) and the median infusion volume per site (39.5 mL/site [range: 6.4–76.0 mL/site]) were higher compared with those of the European study [12].

Of 141 patients screened from the original trial populations, 113 patients completed the patient-reported–outcomes assessments at the end of the pre-Ig20Gly period and fulfilled inclusion criteria for this analysis. The combined analysis of LQI scores included 110 patients from the North American (*n* = 67) and European (*n* = 43) studies who also completed the assessment at the end of the study. All patients from the North American study who met the inclusion criteria provided complete data for the TSQM-9 (*n* = 68). Likewise, all patients from the European study who met inclusion criteria provided complete data for the treatment-preference questionnaire (*n* = 45).

Demographics and baseline clinical characteristics for the 113 patients from the combined study sample are shown in Table 2. Male patients and adults aged ≥18 years comprised more than half (58.4% each) of the combined study sample (*N* = 113). Most patients were white (93.8%) and of non-Latino/Hispanic ethnicity (95.6%). Common variable immune deficiency was the most common PID diagnosis (42.5%), followed by specific antibody deficiency (30.1%) and agammaglobulinemia (18.6%). Before study entry, 76 (67.3%) patients were treated with IVIG, most (*n* = 64, 84.2%) of whom were infused outside the home. Nearly all patients on prior SCIG (91.9%; 34/37) had infused at home.

**Table 2:** Patient demographics and baseline clinical and treatment characteristics

Characteristic, n (%)	All patients			European study			North American study		
	All (***N*** = 113)	Treatment before study		All (***n*** = 45)	Treatment before study		All (***n*** = 68)	Treatment before study	
		IVIG (***n*** = 76)	SCIG (***n*** = 37)		IVIG (***n*** = 30)	SCIG (***n*** = 15)		IVIG (***n*** = 46)	SCIG (***n*** = 22)
Age, y									
0–12	29 (25.7)	20 (26.3)	9 (24.3)	14 (31.1)	12 (40.0)	2 (13.3)	15 (22.1)	8 (17.4)	7 (31.8)
13–17	18 (15.9)	14 (18.4)	4 (10.8)	9 (20.0)	7 (23.3)	2 (13.3)	9 (13.2)	7 (15.2)	2 (9.1)
≥ 18	66 (58.4)	42 (55.3)	24 (64.9)	22 (48.9)	11 (36.7)	11 (73.3)	44 (64.7)	31 (67.4)	13 (59.1)
Sex									
Female	47 (41.6)	31 (40.8)	16 (43.2)	16 (35.6)	10 (33.3)	6 (40.0)	31 (45.6)	21 (45.7)	10 (45.5)
Male	66 (58.4)	45 (59.2)	21 (56.8)	29 (64.4)	20 (67.7)	9 (60.0)	37 (54.4)	25 (54.3)	12 (54.5)
Race									
Asian	2(1.8)	1 (1.3)	1 (2.7)	1 (2.2)	0 (0.0)	1 (6.7)	1 (1.5)	1 (2.2)	0 (0.0)
Black	3 (2.7)	2 (2.6)	1 (2.7)	0 (0.0)	0 (0.0)	0 (0.0)	3 (4.4)	2 (4.3)	1 (4.5)
Other	2 (1.8)	0 (0.0)	2 (5.4)	0 (0.0)	0 (0.0)	0 (0.0)	2 (2.9)	0 (0.0)	2 (9.1)
White	106 (93.8)	73 (96.1)	33 (89.2)	44 (97.8)	30 (100.0)	14 (93.3)	62 (91.2)	43 (93.5)	19 (86.4)
Ethnicity									
Hispanic or Latino	5 (4.4)	5 (6.6)	0 (0.0)	0 (0.0)	0 (0.0)	0 (0.0)	5 (7.4)	5 (10.9)	0 (0.0)
Non-Latino/Hispanic	108 (95.6)	71 (93.4)	37 (100.0)	45 (100.0)	30 (100.0)	15 (100.0)	63 (92.6)	41 (89.1)	22 (100.0)
Country									
Austria	1 (0.9)	0 (0.0)	1 (2.7)	1 (2.2)	0 (0.0)	1 (6.7)	–	–	–
Belgium	1 (0.9)	1 (1.3)	0 (0.0)	1 (2.2)	1 (3.3)	0 (0.0)	–	–	–
Canada	2 (1.8)	2 (2.6)	0 (0.0)	–	–	–	2 (2.9)	2 (4.3)	0 (0.0)
Germany	12 (10.6)	6 (7.9)	6 (16.2)	12 (26.7)	6 (20.0)	6 (40.0)	–	–	–
Hungary	20 (17.7)	19 (25.0)	1 (2.7)	20 (44.4)	19 (63.3)	1 (6.7)	–	–	–
Netherlands	2 (1.8)	1 (1.3)	1 (2.7)	2 (4.4)	1 (3.3)	1 (6.7)	–	–	–
Sweden	3 (2.7)	1 (1.3)	2 (5.4)	3 (6.7)	1 (3.3)	2 (13.3)	–	–	–
United Kingdom	6 (5.3)	2 (2.6)	4 (10.8)	6 (13.3)	2 (6.7)	4 (26.7)	–	–	–
United States	66 (58.4)	44 (57.9)	22 (59.5)	–	–	–	66 (97.1)	44 (95.7)	22 (100.0)
Site of care									
Home	46 (40.7)	12 (15.8)	34 (91.9)	18 (40.0)	3 (10.0)	15 (100.0)	28 (41.2)	9 (19.6)	19 (86.4)
Other	67 (59.3)	64 (84.2)	3 (8.1)	27 (60.0)	27 (90.0)	0 (0.0)	40 (58.8)	37 (80.4)	3 (13.6)
PID diagnosis									
Common variable immune deficiency (including familial TACI mutation C.512 > G)	48 (42.5)	33 (43.4)	15 (40.5)	28 (62.2)	18 (60.0)	10 (66.7)	20 (29.4)	15 (32.6)	5 (22.7)
Congenital agammaglobulinemia - X-linked or autosomal recessive	21 (18.6)	13 (17.1)	8 (21.6)	10 (22.2)	6 (20.0)	4 (26.7)	11 (16.2)	7 (15.2)	4 (18.2)
Hyper-IgM – X-linked or autosomal recessive	5 (4.4)	5 (6.6)	0 (0.0)	3 (6.7)	3 (10.0)	0 (0.0)	2 (2.9)	2 (4.3)	0 (0.0)
IgG subclass deficiency – isolated, or with low IgG	1 (0.9)	0 (0.0)	1 (2.7)	0 (0.0)	0 (0.0)	0 (0.0)	1 (1.5)	0 (0.0)	1 (4.5)
IgM and IgG deficiencies	1 (0.9)	0 (0.0)	1 (2.7)	1 (2.2)	0 (0.0)	1 (6.7)	0 (0.0)	0 (0.0)	0 (0.0)
Severe combined immunodeficiency	1 (0.9)	0 (0.0)	1 (2.7)	0 (0.0)	0 (0.0)	0 (0.0)	1 (1.5)	0 (0.0)	1 (4.5)
Specific antibody deficiency – isolated, or with hypogammaglobulinemia or IgG subclass deficiency	36 (31.9)	25 (32.9)	11 (29.7)	3 (6.7)	3 (10.0)	0 (0.0)	33 (48.5)	22 (47.8)	11 (50.0)

*Ig* immunoglobulin, *IVIG* intravenous immunoglobulin, *PID* primary immunodeficiency diseases, *SCIG* subcutaneous immunoglobulin, *TACI* transmembrane activator and calcium-modulating cyclophilin ligand interactor

### Changes in LQI scores

#### Combined analysis

**Fig. 1:**
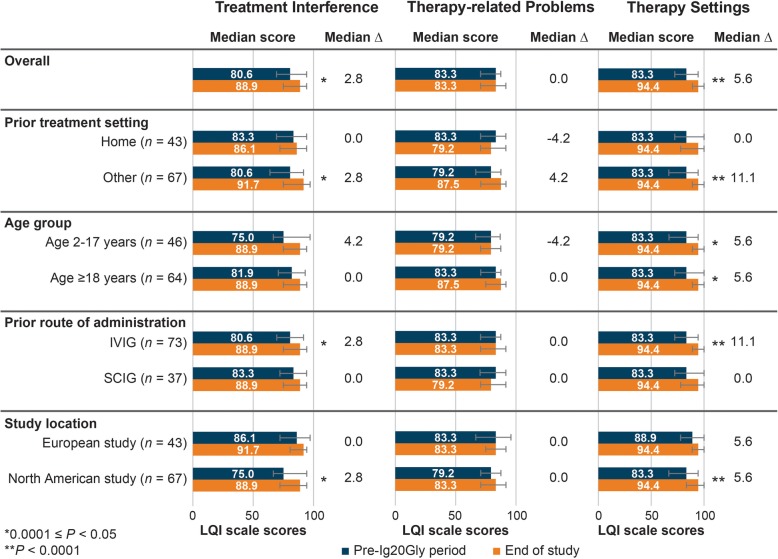
Median LQI domain scale scores (range: 0–100) from combined analysis of data from the North American and European studies from pre-Ig20Gly period to the end of the study for Ig20Gly therapy by prior treatment setting, age group, prior route of administration, and study location. Ig20Gly, immune globulin subcutaneous (human) 20%; IVIG, intravenous immunoglobulin; LQI, Life Quality Index; SCIG, subcutaneous immunoglobulin. Note: Bar charts indicate the median score and interquartile range (error bar) for each sample or subgroup in the pre-Ig20Gly period and at the end of the study. The median ∆ value is the median within-patient difference in scores between the end of the pre-Ig20Gly period and the end of the study. *P* values indicate statistical significance for differences in distributions of scores between the pre-Ig20Gly period and the end of the study assessed using the Wilcoxon signed-rank test

Median LQI scores for the pre-Ig20Gly period and end of study, *P* values for the Wilcoxon signed-rank test of the distribution, and within-patient median ∆ values for the combined analysis (*n* = 110) are shown in Fig. 1. Overall within-patient scores improved significantly for the *treatment interference* (median ∆: + 2.8; *P* = 0.006) and *therapy setting* domains (median ∆: + 5.6; *P* < 0.0001). Significant within-patient differences were observed for those who previously received treatment outside the home (*treatment interference*: median ∆: + 2.8, *P* = 0.001; *therapy setting*: median ∆: + 11.1; *P* < 0.0001), previously received IVIG (*treatment interference*: median ∆: + 2.8, *P* = 0.003; median ∆: + 11.1, *therapy setting*: *P* < 0.0001), and were in the North American study (*treatment interference*: median ∆: + 2.8, *P* = 0.013; *therapy setting*: median ∆: + 5.6, *P* < 0.0001). For both patients aged 2–17 and ≥18 years, the within-patient difference in *therapy settings* scores was significant (median ∆: + 5.6, *P =* 0.001).

**Fig. 2:**
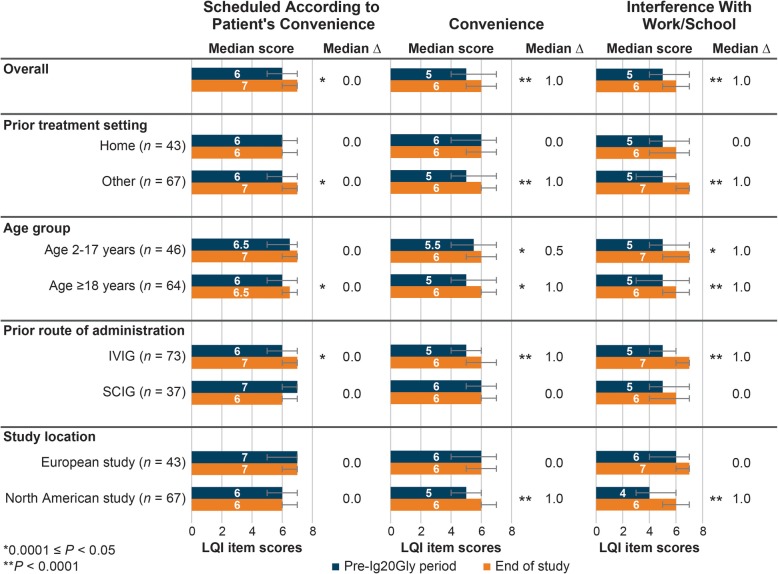
Median LQI selected item scores (range: 0–8) from combined analysis of data from the North American and European studies from pre-Ig20Gly period to end of study for Ig20Gly therapy by prior treatment setting, age group, prior route of administration, and study location. Ig20Gly, immune globulin subcutaneous (human) 20%; IVIG, intravenous immunoglobulin; LQI, Life Quality Index; SCIG, subcutaneous immunoglobulin. Note: Bar charts indicate the median score and interquartile range (error bars) for each sample or subgroup in the pre-Ig20Gly period and at the end of the study. The median ∆ value is the median within-patient difference in scores between the end of the pre-Ig20Gly period and the end of the study. *P* values indicate statistical significance for differences in distributions of scores between the pre-Ig20Gly period and the end of the study assessed using the Wilcoxon signed-rank test

Figure 2 shows the scores for selected LQI items for the overall population. Statistically significant within-patient differences from the pre-Ig20Gly period to the end of the study period were observed for *convenience* (*P* < 0.0001), *scheduled according to patient’s convenience* (*P* = 0.011), and *interference with work/school* (*P* < 0.0001); median ∆ values were + 1.0, 0.0, and + 1.0, respectively. Statistically significant within-patient differences were observed for patients who were previously treated outside the home, aged ≥18 years, and previously treated with IVIG for all selected item scores.

#### North American study

At the end of the Ig20Gly treatment period, patients in the North American study (*n* = 67; 1 patient did not complete the LQI) perceived significantly greater satisfaction in the *treatment interference* (*P* = 0.013) and *therapy setting* (*P* < 0.0001) domains compared with the pre-Ig20Gly period (Fig. 1); median within-patient ∆ values were + 2.8 and + 5.6, respectively. In the *therapy setting* domain, patients reported significant improvement both among those who switched from prior IVIG (*P* < 0.0001) and those previously receiving SCIG (*P* = 0.005); median within-patient ∆ values were + 11.1 and + 5.6, respectively. However, results were not statistically different in the *treatment interference* and *therapy-related problems* domains between those who previously received IVIG and SCIG (Additional file Figure S1).

At the end of the Ig20Gly treatment period, significantly improved scores were observed for the selected LQI items *convenience* (*P* < 0.0001) and *interference with work/school* (*P* < 0.0001) LQI item scores (Fig. 2). Median within-patient ∆ values were + 1.0 for both *convenience* and *interference with work/school*. Improvements for these items were observed regardless of prior route of administration (Additional file Figure S1). Satisfaction with scheduling treatment according to the patient’s convenience did not improve significantly in the total population or after stratifying by the prior route of administration (Additional file Figure S1).

#### European study

Patients in the European study (*n* = 43) showed no significant changes in any of the median domain and item scores at the end of the study compared with the pre-Ig20Gly period (Figs. 1 and 2). However, patients who switched from IVIG (*n* = 28) perceived statistically significant improvements after treatment with Ig20Gly in the *treatment interference* (*P* = 0.018) and *therapy setting* (*P* = 0.005) domains; median within-patient ∆ values were + 4.2 and + 11.1, respectively (Additional file Figure S2). Significant within-patient improvements were also observed for the LQI items assessing *scheduled according to patient’s convenience* (median ∆: 0.0; *P* = 0.004) and *interference with work/school* (median ∆: + 1.0; *P* = 0.003); median ∆ values were 0.0 and + 1.0, respectively (Additional file Figure S2). Patients who previously received SCIG reported no significant changes.

### Changes in TSQM-9 (North American study)

**Fig. 3:**
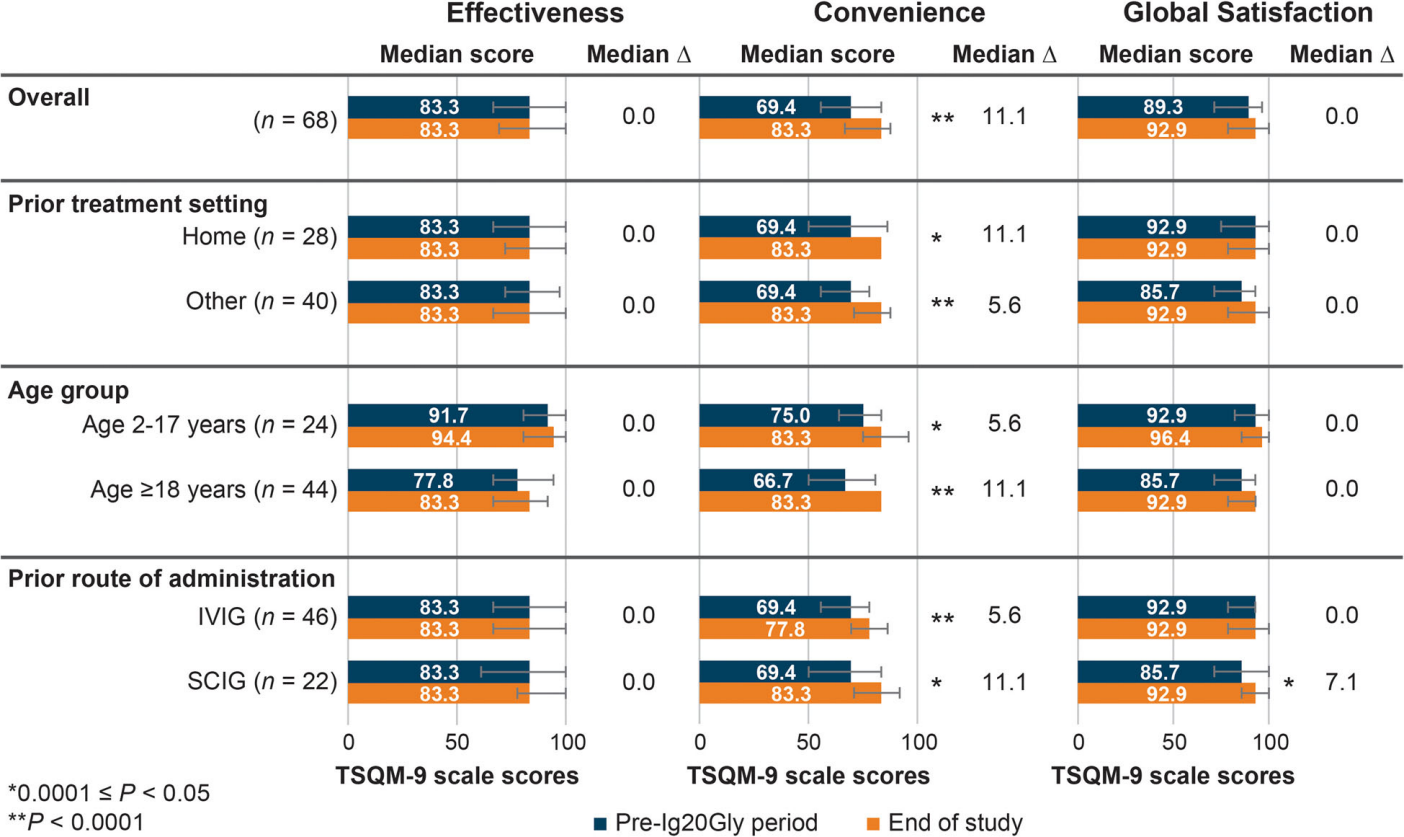
Median TSQM-9 domain scale scores (range: 0–100) from the North American study from pre-Ig20Gly period to the end of the study for Ig20Gly therapy by prior treatment setting, age group, and prior route of administration. Ig20Gly, immune globulin subcutaneous (human) 20%; IVIG, intravenous immunoglobulin; SCIG, subcutaneous immunoglobulin; TSQM-9, Treatment Satisfaction Questionnaire for Medication-9.Note: Bar charts indicate the median score and interquartile range (error bars) for each sample or subgroup in the pre-Ig20Gly period and at the end of the study. The median ∆ value is the median within-patient difference in scores between the end of the pre-Ig20Gly period and the end of the study. *P* values indicate statistical significance for differences in distributions of scores between the pre-Ig20Gly period and the end of the study assessed using the Wilcoxon signed-rank test

Figure 3 shows median TSQM-9 scores for the pre-Ig20Gly period and end of study, *P* values for the Wilcoxon signed-rank test of the distribution, and within-patient median ∆ values from the North American study. At the end of the Ig20Gly treatment period, patients (*n* = 68) reported significantly higher scores in the *convenience* domain (median within-patient ∆: + 11.1; *P* < 0.0001). *Convenience* domain scores improved significantly regardless of prior route of administration, age group, or prior treatment setting. Significantly significant within-patient improvements in *global satisfaction* were observed only for patients who previously received SCIG (median ∆: + 7.1; *P* = 0.001). There was no statistically significant difference in the *effectiveness* domain overall or for any subgroup (Fig. 3).

**Fig. 4:**
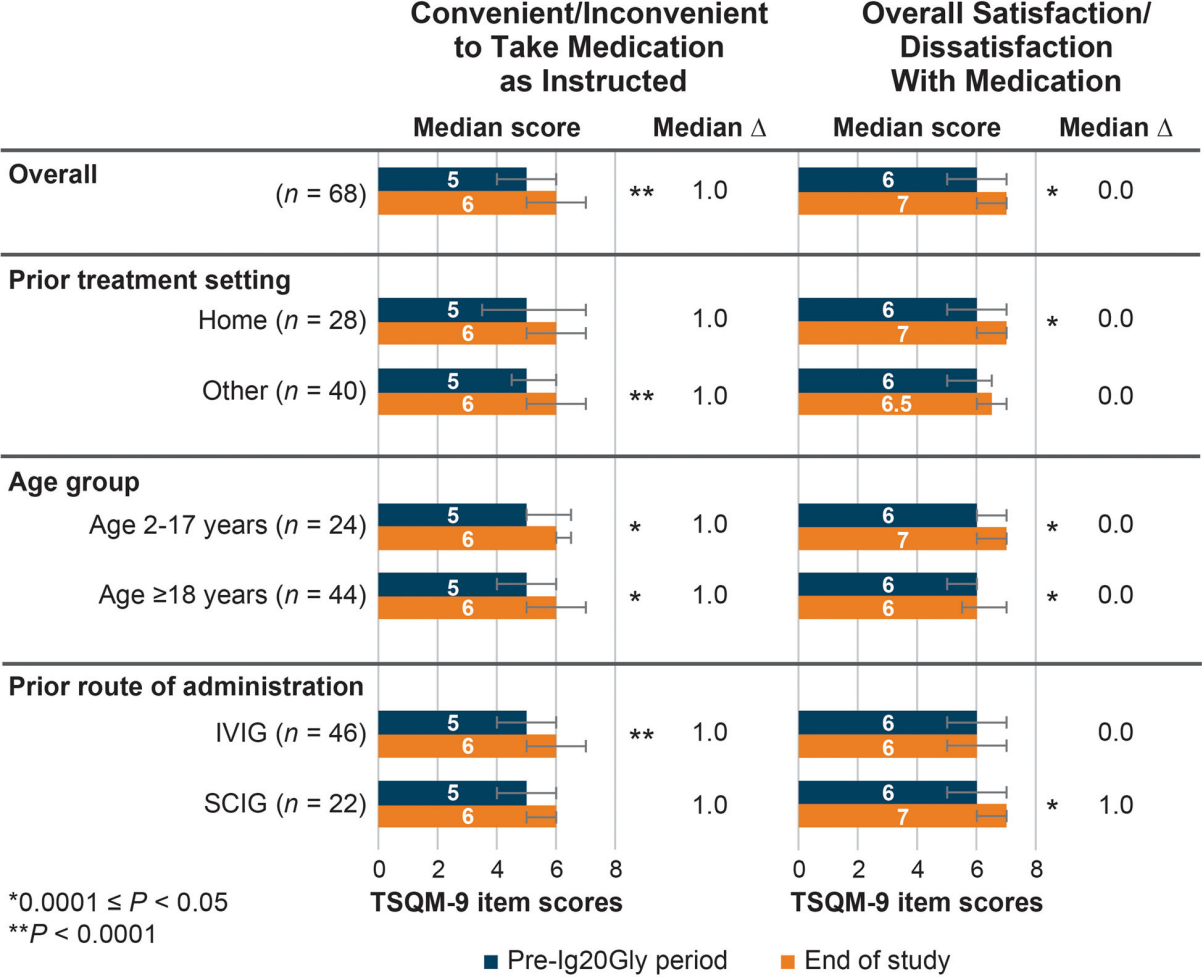
Median TSQM-9 selected item scores (range: 0–8) from the North American study from pre-Ig20Gly period to the end of the study for Ig20Gly therapy by prior treatment setting, age group, and prior route of administration. Ig20Gly, immune globulin subcutaneous (human) 20%; IVIG, intravenous immunoglobulin; SCIG, subcutaneous immunoglobulin; TSQM, Treatment Satisfaction Questionnaire for Medication. Note: Bar charts indicate the median score and interquartile range (error bars) for each sample or subgroup in the pre-Ig20Gly period and at the end of the study. The median ∆ value is the median within-patient difference in scores between the end of the pre-Ig20Gly period and the end of the study. *P* values indicate statistical significance for differences in distributions of scores between the pre-Ig20Gly period and the end of the study assessed using the Wilcoxon signed-rank test

TSQM-9 item level scores are shown in Fig. 4. Significant within-patient increases in scores for *convenience of taking medication as instructed* (median ∆: + 1.0; *P* < 0.0001) and *overall satisfaction/dissatisfaction with medication* (median ∆: 0.0; *P* = 0.004) were observed. Item-level scores for the *convenience of taking medication as instructed* significantly improved for those who were previously treated outside the home (*P* < 0.0001), those who were previously treated with IVIG (*P* < 0.0001), and for patients who were aged 2–17 (*P* = 0.018) and ≥18 years (*P* = 0.0002). Median within-patient ∆ values were + 1.0 for patients who were previously treated outside the home, those who were previously treated with IVIG and both age groups. The distribution of the item-level scores (Fig. 4) for *overall satisfaction/dissatisfaction with medication* improved significantly for patients previously treated at home, those previously treated with SCIG, and both age groups.

### Treatment preference (European study)

**Fig. 5:**
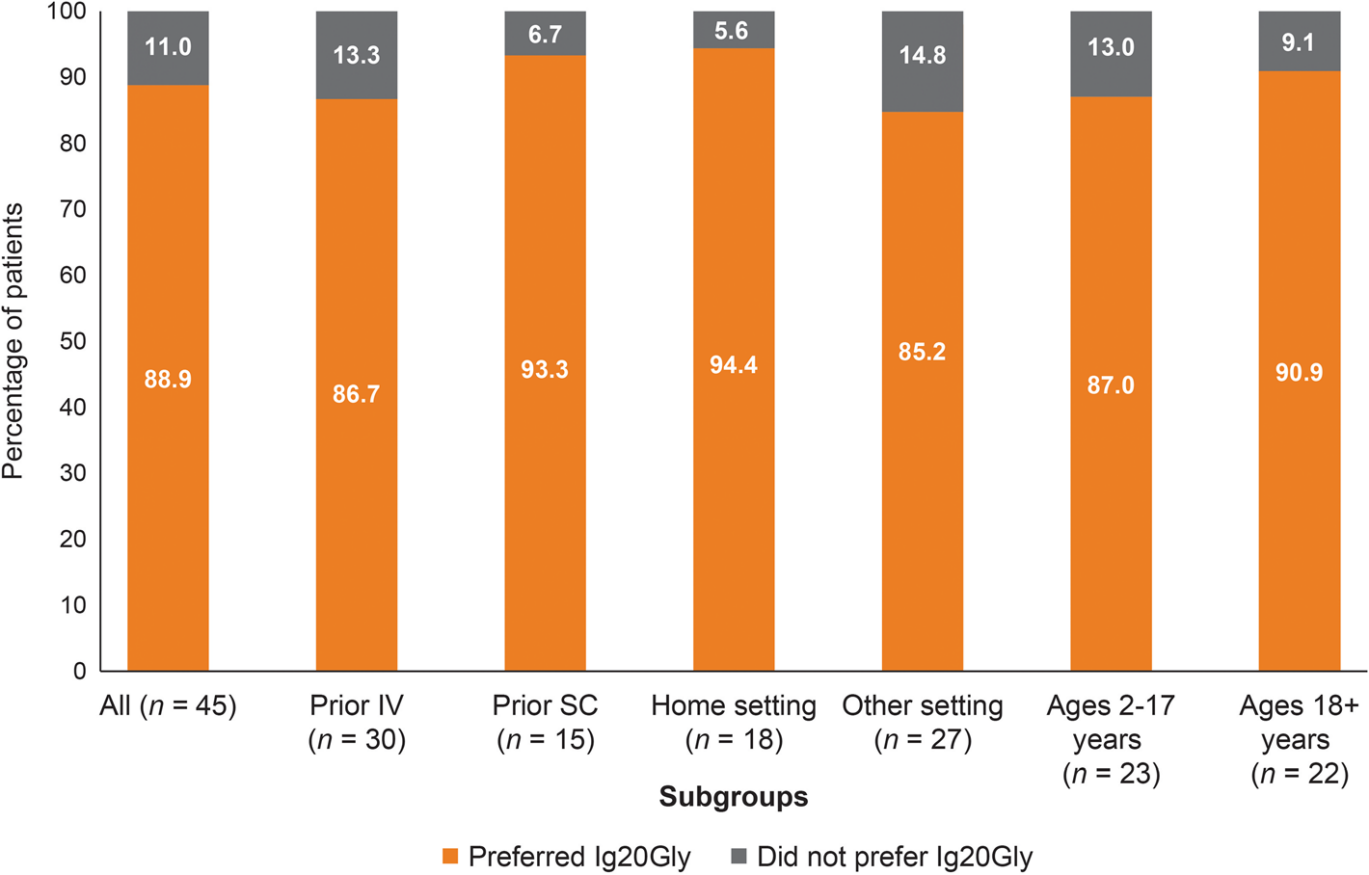
Treatment preference to continue Ig20Gly treatment in the European study. Ig20Gly, immune globulin subcutaneous (human) 20%; IV, intravenous; SC, subcutaneous

Overall, 88.9% (40/45) of patients in the European study stated that they would prefer to continue Ig20Gly therapy over other treatments. All subgroups had similarly high proportions of patients favoring continuation of Ig20Gly treatment (Fig. 5). The patients also preferred to administer in the home (88.9%; 40/45), although only one-third (33.3%) had done so previously (data not shown in figure).

In the European study, the attributes most strongly preferred by patients (based on the percentages of “like” or “like very much” responses) included the ability to fit the treatment into his or her own schedules (95.6%) and the ability to administer without supervision (95.6%), overall convenience (93.3%), the potential to self-administer (91.1%), and ease of administration (91.1%) (Additional file Figure S3). Attributes that were most commonly “disliked/disliked very much” included the frequency of administration (15.5%) and the number of needle sticks per month (13.3%) The subgroup analyses demonstrated similar trends in attribute-preference ratings (data not shown).

## Discussion

Over a dozen IGRT products are available in North America and Europe, which vary with respect to IG concentration, infusion frequency, route of administration, and other considerations [25]. In a recent meta-analysis, IGRT products were equally effective for preventing infections in patients who have PID, regardless of the route of administration [26]. In terms of safety and tolerability, patients receiving SCIG therapies generally report localized infusion-site reactions, whereas those receiving IVIG therapies are more likely to experience systemic adverse reactions [27–29]. Therefore, treatment decisions are greatly influenced by factors, including tolerability and patient preference.

This analysis evaluated various aspects of the patient experience with Ig20Gly therapy in North America and Europe. The LQI indicated improvements in satisfaction related to *treatment interference* and *therapy setting* in the overall population; however, the analysis found some differences in the reported experiences of the subpopulations. Patients perceived improved convenience of Ig20Gly treatment compared with previous therapy as measured by the TSQM-9. After ≥11 months on Ig20Gly therapy, the European study found that 89% of patients wanted to continue, with similar findings across all subgroups, including those patients who had been previously treated with another SCIG. Overall, the combined findings from the LQI, TSQM-9, and treatment-preference surveys indicated that patients had improved treatment experience after Ig20Gly treatment compared with previous therapies.

The combined LQI analyses confirmed the findings of the North American study, which showed that patients who switched from IVIG to Ig20Gly treatment for approximately 1 year were significantly more satisfied with respect to the *interference* (*interference with work or school* item), *therapy settings* (*therapy settings* domain), and *convenience* (*convenience* item) aspects of treatment. Although the European study reported more modest changes, patients in the European study had generally higher baseline LQI scores than those in the North American study, suggesting that the former population may have been relatively more satisfied before initiating Ig20Gly treatment. One-third (15/45) of the patients in the European study had switched from prior SCIG treatment. Given that these patients had received SCIG in both periods 1 and 2, higher baseline scores and smaller effects were hypothesized. This apparent “ceiling effect” may have also accounted for subgroup differences in the combined analysis. Patients treated previously at home or with prior SCIG tended to report greater satisfaction at baseline compared with those switching from other settings or IVIG. Therefore, patients having prior IVIG (but not prior SCIG) treatment perceived significant improvement in the *treatment interference* domain, whereas patients treated previously in other settings, but not at home, were significantly more satisfied in the *therapy setting* domain. Similarly, item-level LQI scores in the combined analyses did not increase significantly for patients continuing treatment at home.

Overall, the present LQI findings are consistent with the results of several clinical trials and real-world studies, which indicated that SCIG treatment or treatment in the home had higher reported satisfaction compared with IVIG or hospital-based treatment [21, 30]. Mean scores for all LQI domains increased significantly from baseline to 12 weeks among patients who switched to home administration of 20% SCIG IgPro20 (Hizentra, CSL Behring AG, Bern, Switzerland) from IVIG or other SCIG in 2 phase 3 clinical studies [31]. In the prospective, observational, French cohort “Visages” study (*N* = 116) [21], satisfaction measured in the LQI *therapy setting* domain was significantly higher for home-based SCIG compared with hospital-based IVIG and satisfaction in the *treatment interference* domain was significantly higher for home-based SCIG compared with home-based IVIG; however, hospital-based IVIG and home-based IVIG had no differential effects. The route of administration and site of treatment had no significant impact on satisfaction concerning *therapy-related problems* [21]. The US IDEaL (Immunoglobulin Diagnosis, Evaluation, and Key Learnings) patient registry survey [30] found that after 12 months of home-based SCIG (80%) or home-based IVIG (20%) treatment, 92% (108/118) of respondents reported positive views regarding treatment convenience. Although the LQI *convenience* scores did not differ between the groups, over time, these positive perceptions decreased with IVIG and increased with SCIG treatment [30]. In addition, several nonrandomized prospective studies conducted in Europe have similarly reported improvements in LQI scores for patients who switched from hospital-based IVIG to home-based SCIG treatment [15, 24, 32].

The TSQM-9 responses in the present analysis showed that patients in the North American study valued the convenience of home-based Ig20Gly therapy, regardless of prior route of administration, age group, or prior treatment setting, based on the responses to the overall *convenience* domain. The *global satisfaction* domain scores improved significantly only in patients on prior SCIG; however, on the specific item *“overall satisfaction/dissatisfaction with medication,*” most subgroups, except patients previously treated with IVIG and outside the home, had higher score distributions. The difference between the results in the *global satisfaction* domain score and the overall satisfaction item may be because the domain also includes confidence and certainty regarding the positive aspects of treatment. In contrast to the LQI findings, the TSQM-9 scores by domain prior to treatment with Ig20Gly were similar across subgroups defined by previous route of administration and previous treatment setting, but not by age group.

The TSQM was administered in 2 other studies of IG switching [33, 34]. In those studies, switching patients from SCIG 16% (Vivaglobin, CSL Behring GmbH, Marburg, Germany) to a 20% SCIG IgPro20 (Hizentra, CSL Behring AG, Bern, Switzerland), no statistically significant improvements were found [33, 34]. However, several differences between these IgPro20 studies and the present study of Ig20Gly should be noted: The IgPro20 treatment was shorter (24 weeks) than in the studies used in the present analysis, and Ig20Gly and IgPro20 treatments differed in infusion characteristics, with the former allowing for fewer infusion sites and faster infusion rates, leading to shorter infusion times [33, 34]. Similar to the convenience benefits of Ig20Gly therapy observed in the present study, study participants in the IgPro20 study were more satisfied with the frequency and scheduling of infusions after using the 20% SCIG treatment [33, 34].

Patients in the present Ig20Gly European study most strongly preferred treatment aspects that enabled more control and self-administration, which are attributes generally ascribed to home-based SCIG therapy. These preferences mirrored prior observations of the 2011 International Patient Organisation for Primary Immunodeficiencies survey (*N* = 300), which found that SCIG respondents significantly preferred self-administration compared with an appointment with a healthcare provider (*P* < 0.05) [35]. IVIG respondents significantly favored once-monthly treatment compared with more frequent treatments and a single needle stick per infusion compared with 2 or 3 needle sticks. However, both groups significantly preferred home-based therapy to clinic-based options (*P* < 0.05) [35].

This analysis has several limitations; the primary limitation is that the data were collected in an investigational setting, which may not reflect real-world treatment experiences. The source studies [11, 12] included patients from many countries and sites, with potentially differing underlying clinical characteristics and dissimilar practice patterns, both of which may influence the patient experience. A stratified analysis by site was not conducted because the subgroups would have been too small for meaningful statistical analysis. A *P* value correction method for multiple comparisons, such as Bonferroni adjustment, was not used in this analysis; however, for most comparisons, using such a correction method would not have changed the statistical significance of the results. Finally, because the TSQM-9 and the treatment-preference questionnaire were not administered in both studies, we could not combine these results across the European and North American studies. Future studies evaluating the patient experience in real-world settings will expand the evidence base for improving the patient experience among those treated with IG for PID.

## Conclusion

Selection of an IG product and route of administration should consider a wide range of clinical and patient parameters. At the appropriate dosing regimen, SCIG is as efficacious as IVIG, while offering potential advantages, such as the ability to self-administer at home, fewer systemic adverse reactions, higher serum IgG trough levels, and improvement in the patient experience. The choice between IG treatments and, ultimately, achievement of treatment success may depend on the patient’s expectations, preferences, and satisfaction with treatment. Prescribers can consider high levels of patient-reported satisfaction, patient convenience, and preference for Ig20Gly therapy when selecting an appropriate IG treatment option for their patients with PID.

## Supplementary information

**Additional file 1 **: **Figure S1.** LQI Domain and Item Subgroup Scores by Prior Route of Administration From the North American Study (*N* = 67)

**Additional file 2 **: **Figure S2.** LQI Domain and Item Subgroup Scores by Prior Route of Administration From the European Study (*N* = 43)

**Additional file 3 **: **Figure S3.** Preference for Treatment Attributes in the European Study

## Data Availability

The datasets, including the redacted study protocols, redacted statistical analysis plans, and individual participants data supporting the results reported in this article, will be available 3 months after the submission of a request, to researchers who provide a methodologically sound proposal. The data will be provided after its de-identification, in compliance with applicable privacy laws, data protection and requirements for consent and anonymization. Please contact Lisa Meckley, the corresponding author of this study, to request the data.

## Abbreviations

HRQoL: Health-related quality of life
IDEaL: Immunoglobulin Diagnosis, Evaluation, and Key Learnings
IG: Immunoglobulin
Ig20Gly: CUVITRU, 20% subcutaneous immunoglobulin product
IgG: Immunoglobulin G
IgPro20: Hizentra, 20% subcutaneous immunoglobulin product
IGRT: Immunoglobulin replacement therapy
IV: Intravenous
IVIG: Intravenous immunoglobulin
LQI: Life Quality Index
PID: Primary immunodeficiency diseases
SBI: Serious bacterial infection
SC: Subcutaneous
SCIG: Subcutaneous immunoglobulin
TSQM-9: Treatment Satisfaction Questionnaire for Medication-9
