# Supplementary figures and images for: Patient experience with subcutaneous immunoglobulin 20%, Ig20Gly, for primary immunodeficiency diseases: a prespecified post hoc analysis of combined data from 2 pivotal trials
Source: BMC Immunol. 2020 May 4;21:24. doi: 10.1186/s12865-020-00346-z (PMC7197164; doi:10.1186/s12865-020-00346-z)

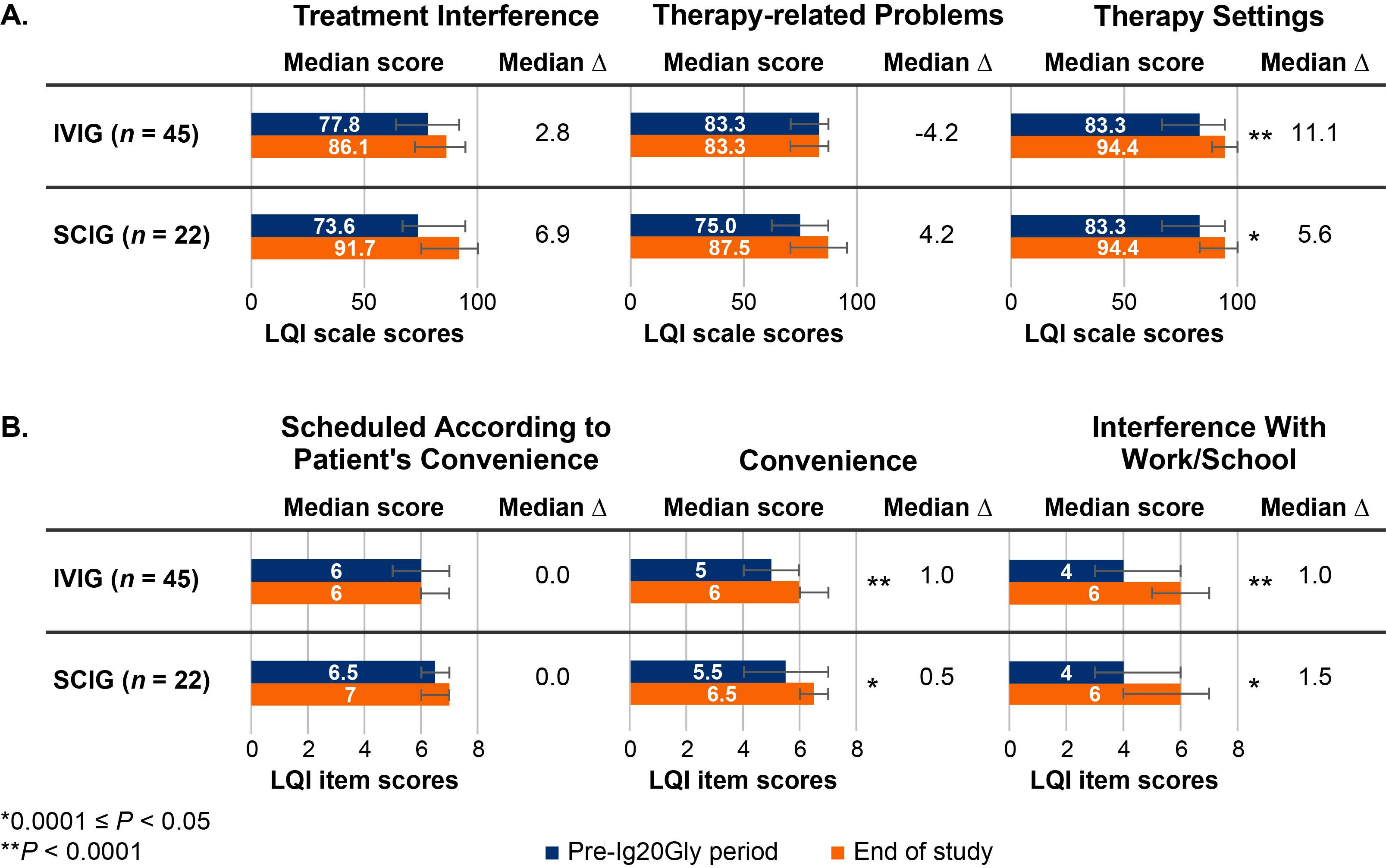

Supplement: Supplementary file 1 — Additional file 1 : Figure S1. LQI Domain and Item Subgroup Scores by Prior Route of Administration From the North American Study (N = 67) [file 12865_2020_346_MOESM1_ESM.jpg]

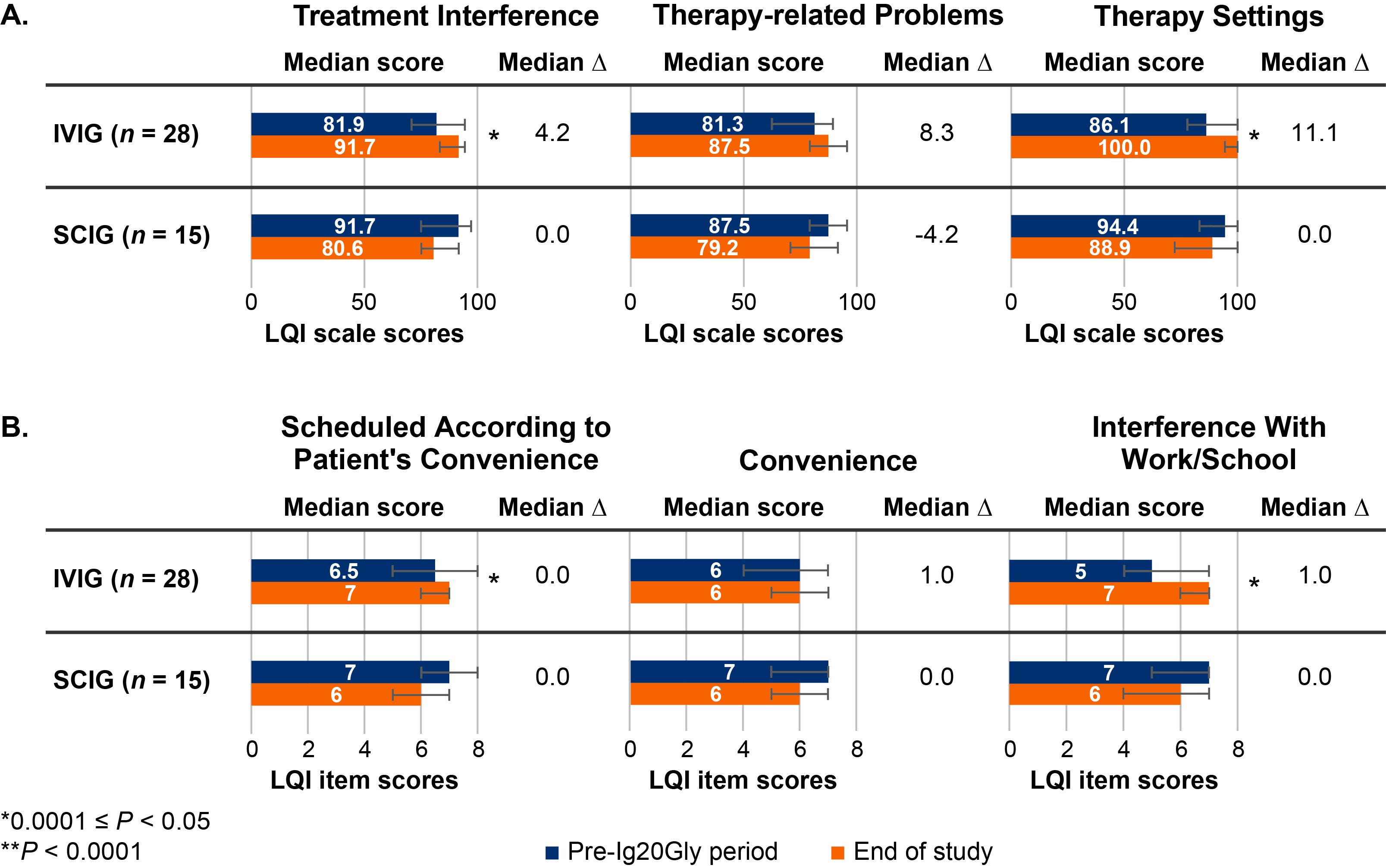

Supplement: Supplementary file 2 — Additional file 2 : Figure S2. LQI Domain and Item Subgroup Scores by Prior Route of Administration From the European Study (N = 43) [file 12865_2020_346_MOESM2_ESM.jpg]

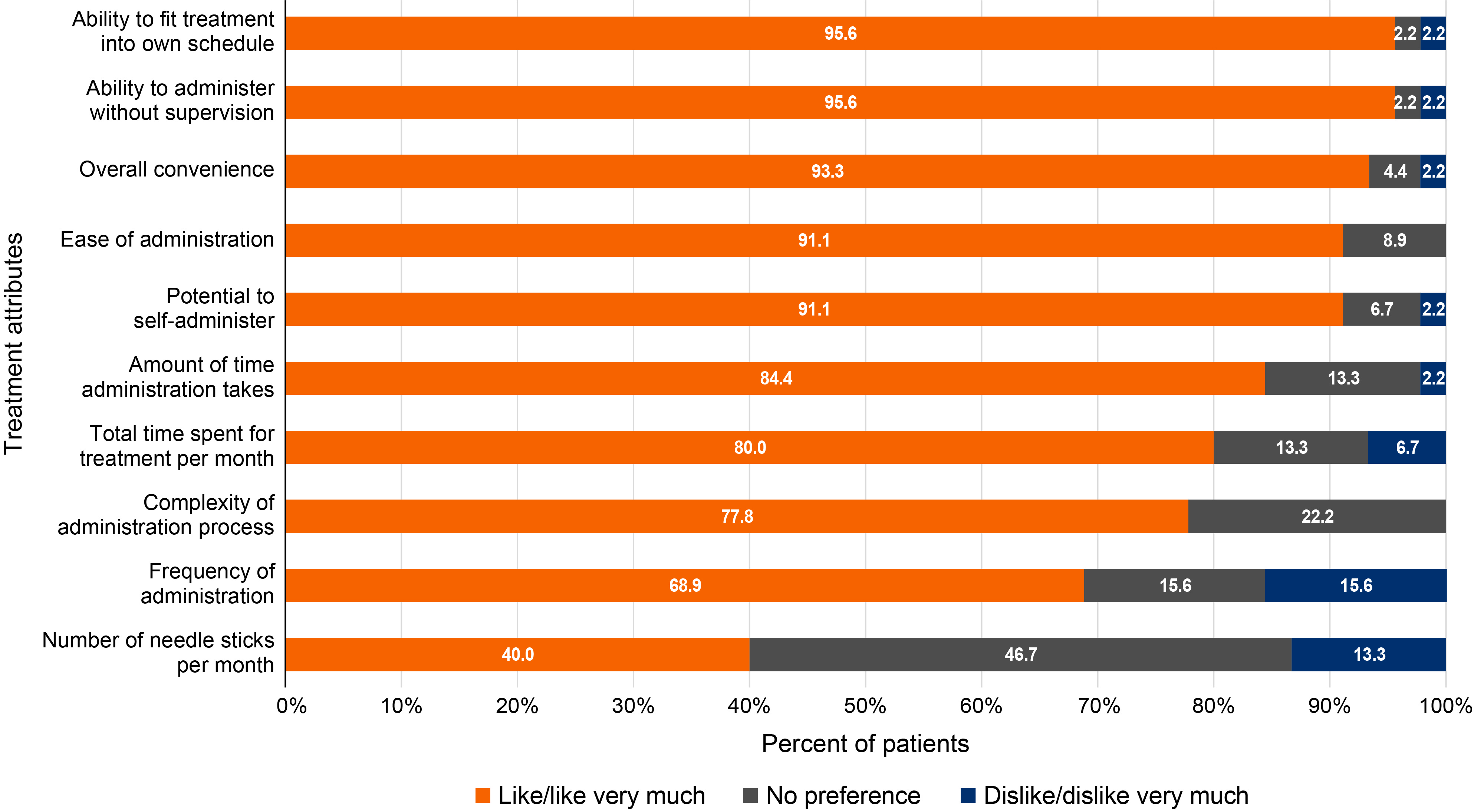

Supplement: Supplementary file 3 — Additional file 3 : Figure S3. Preference for Treatment Attributes in the European Study [file 12865_2020_346_MOESM3_ESM.jpg]
